# Potent Oral Hypoglycemic Agents for Microvascular Complication: Sodium-Glucose Cotransporter 2 Inhibitors for Diabetic Retinopathy

**DOI:** 10.1155/2018/6807219

**Published:** 2018-12-05

**Authors:** Eun Hyung Cho, Se-Jun Park, Seongwook Han, Ji Hun Song, Kihwang Lee, Yoo-Ri Chung

**Affiliations:** ^1^Kong Eye Hospital, Seoul, Republic of Korea; ^2^Department of Cardiology, GangNeung Asan Hospital, University of Ulsan College of Medicine, Gangneung, Republic of Korea; ^3^Department of Ophthalmology, Ajou University School of Medicine, Suwon, Republic of Korea

## Abstract

The purpose of this study was to investigate the effects of sodium-glucose cotransporter 2 inhibitors (SGLT2i) on the progression of diabetic retinopathy (DR) in patients with type 2 diabetes. The medical records of 21 type 2 diabetic patients who used a SGLT2i and 71 patients with sulfonylurea (control) were reviewed retrospectively. The severity of DR was assessed using the Early Treatment Diabetic Retinopathy Study (ETDRS) scale. Fewer patients who used a SGLT2i than control patients with sulfonylurea showed progression of DR based on ETDRS scale (44% versus 14%, *P* = 0.014). Moreover, treatment with a SGLT2i was associated with a significantly lower risk of DR progression (*P* = 0.021), and this effect remained significant after adjusting for the age, duration of diabetes, initial DR grade, and HbA1c level by propensity score matching (*P* = 0.013). Treatment of type 2 diabetic patients with a SGLT2i slowed the progression of DR compared to sulfonylurea, which is independent of its effect on glycemic control. This study provides a foundation for further evaluation of the effect of SGLT2i on the progression of DR.

## 1. Introduction

The prevalence of type 2 diabetes is dramatically increasing worldwide, and an estimated 592 million people will have this disease by 2035 [1, 2]. Diabetic retinopathy (DR) is one of the major microvascular complications of diabetes and is also the leading cause of blindness among working-age people in developed countries [3, 4]. Reduction of hyperglycemia is the primary goal of most therapies for type 2 diabetes, and these therapies may also prevent or arrest the development of DR [5]. In addition to strict glycemic control, use of systemic agents in other therapeutic classes, such as candesartan and fenofibrate, can delay the progression of DR in patients with type 2 diabetes [6, 7].

The sodium-glucose cotransporter 2 inhibitors (SGLT2i) are a novel class of oral hypoglycemic agents that decrease the reabsorption of glucose in the renal proximal tubules [8, 9]. These agents can reduce the level of serum glycosylated hemoglobin (HbA1c), induce weight loss, and decrease blood pressure [8–10]. Among several SGLT2i, empagliflozin and dapagliflozin are now available in Korea, and clinicians usually recommended its use in combination with other hypoglycemic agents as a second- or third-line therapy for type 2 diabetes [11].

There are recent reports that SGLT2i also reduce macrovascular and microvascular complications by affecting vascular remodeling [12, 13]. This suggests that these drugs have renoprotective effects. Thus, the SGLT2i not only improve glycemic control but also have important hemodynamic and nonhemodynamic effects [14]. Because the pathogenesis of diabetic nephropathy and DR are similar [15], we hypothesized that SGLT2i may also protect against the progression of DR, which is a topic that has not yet been examined. We retrospectively examined the records of patients with type 2 diabetes to determine the effect of SGLT2i on the progression of DR.

## 2. Materials and Methods

### 2.1. Study Population

**Figure 1:**
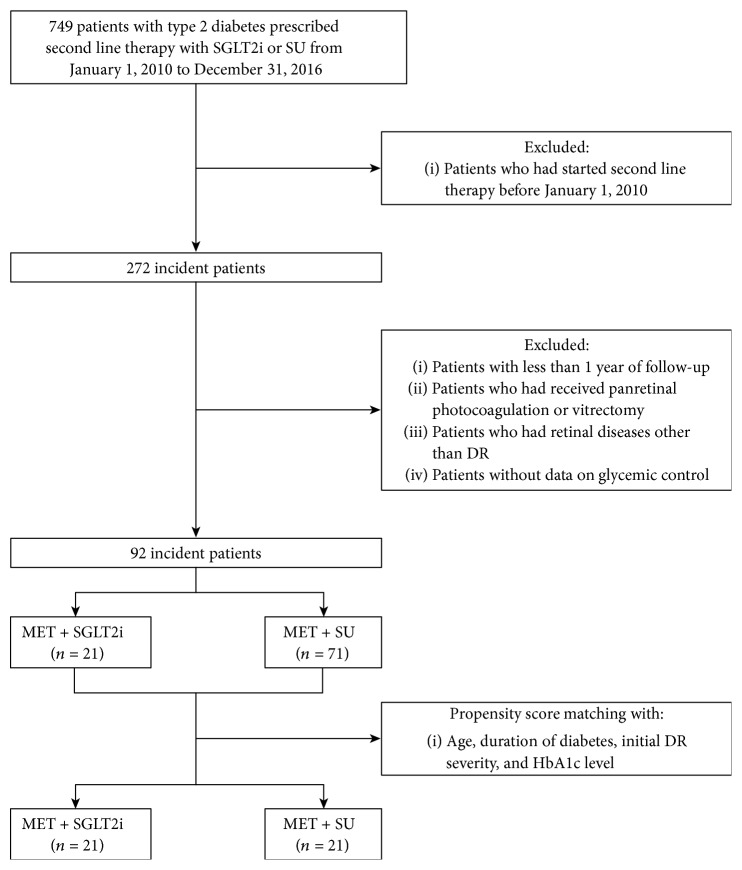
Flow chart of patients included in this study. DR = diabetic retinopathy; MET = metformin; SGLT2i = sodium-glucose cotransporter 2 inhibitor; SU = sulfonylurea.

The medical records of 49 patients with type 2 diabetes who used SGLT2i (SGLT2i group) as add-on medication to metformin and were followed up by the Ophthalmology and Endocrinology Departments of Ajou University Hospital (Suwon, Korea) from January 2010 to December 2016 were retrospectively reviewed (Figure 1). The records of 700 patients with type 2 diabetes who received metformin and sulfonylurea for their diabetes during the same period were also reviewed as control group. Those with dipeptidyl peptidase-4 (DPP4) inhibitors, which may affect DR, were initially excluded from the study [16, 17]. Patients were also excluded if they had (i) no fundus photographs or fluorescein angiography results to grade DR severity, (ii) a history of laser photocoagulation or vitrectomy at initial presentation, (iii) the presence of a retinal pathology other than DR, and (iv) received follow-up for less than 1 year. This study complied with the Declaration of Helsinki and was approved by the Institutional Review Board of Ajou University Hospital (AJIRB-MED-MDB-17-312).

### 2.2. Clinical Parameters

The demographic and clinical characteristics of the patients were obtained from their medical records. In particular, age, sex, duration of type 2 diabetes, prior history of hypertension and cardiovascular diseases (i.e., coronary artery disease or ischemic stroke (or transient ischemic attack)), serum lipid profile, estimated glomerular filtration rate (eGFR), and ophthalmic history (including DR severity and number of intravitreal injections of antivascular endothelial growth factor (VEGF) agents) were recorded. Patients with eGFR less than 60 mL/min/1.73m^2^ were excluded to avoid the effect of renal function.

The severity of DR was assessed using the Early Treatment Diabetic Retinopathy Study (ETDRS) severity scale [18]. The ETDRS severity scale was determined from fundus photographs and simultaneously performed fluorescein angiography at initial presentation and after at least one year of follow-up by the same experienced retinal specialist (Y. R. Chung). DR progression was defined as an increase of 2 or more steps on the ETDRS severity scale during follow-up [19, 20].

### 2.3. Statistics

Categorical variables were compared using the chi-square test, and continuous variables were compared using the independent *t*-test or Mann-Whitney test, depending on the distribution. Statistical analysis were performed using PASW software (version 18.0, SPSS, Chicago, IL), and statistical significance was defined as a *P* value below 0.05.

To adjust for confounding factors in the analysis, 1 : 1 propensity score matching of the SGLT2i and the control groups was performed using logistic regression analysis, with matching for age, duration of diabetes, HbA1c level, and initial ETDRS score. Logistic regression was also used to identify the factors associated with the progression of DR.

## 3. Results

**Figure 2:**
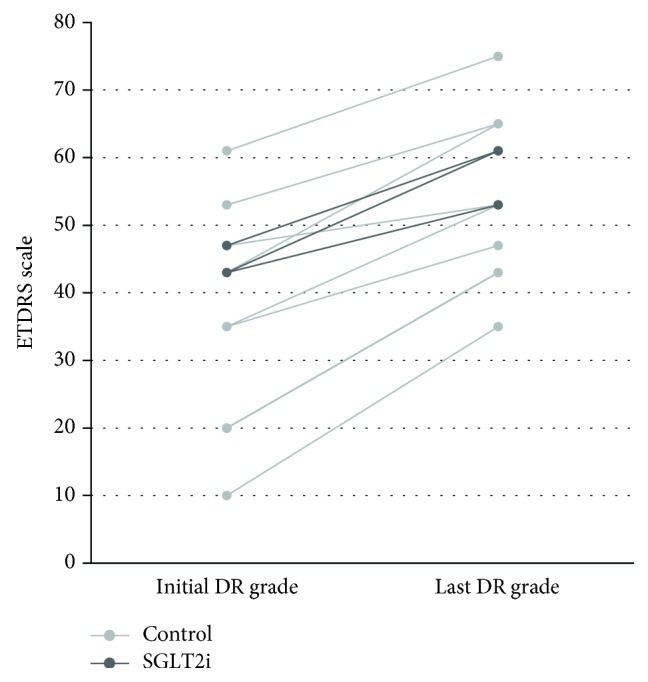
Change of ETDRS scales in patients with DR progression. DR = diabetic retinopathy; ETDRS = Early Treatment Diabetic Retinopathy Study; SGLT2i = sodium-glucose cotransporter 2 inhibitor.

We ultimately enrolled 21 patients in the SGLT2i group and 71 patients in the control group (Table 1). Overall, the mean age was 56.3 ± 12.1 years, 54 (59%) were male, the mean duration of diabetes was 11.4 ± 9.1 years, and the mean follow-up period was 23.9 ± 12.4 months. Three patients in the SGLT2i group took empagliflozin and 18 took dapagliflozin. Patients using SGLT2i was younger than patients in the control group and had higher level of HbA1c. Significantly, fewer patients in the SGLT2i group had DR progression relative to the control group (44% vs. 14%, *P* = 0.014). The change of ETDRS scales in patients with DR progression is shown in Figure 2.

The glycemic control in diabetic patients could possibly affect the rate of DR progression, so differences between the 2 groups at baseline could have affected the results presented in Table 1. Thus, we performed propensity score matching to adjust for factors that could potentially influence DR progression (age, duration of diabetes, glycemic status (HbA1c), and initial DR severity). After propensity score matching (Table 2), the SGLT2i group still showed less progression of DR (*P* = 0.009). The mean number of intravitreal anti-VEGF agent injections and HbA1c levels were not significantly different between the 2 groups in the unmatched analysis (Table 1) and the matched analysis (Table 2).

We performed logistic regression analysis to identify the factors associated with DR progression both in unmatched patients (Table 3) and in matched patients (Table 4). The results show that treatment with a SGLT2i was associated with a significantly lower risk of DR progression (odds ratio (OR) = 0.215, 95% confidence interval (CI) = 0.058–0.796, *P* = 0.021). This significant difference remained after propensity score matching for age, the duration of diabetes, initial DR grade, and HbA1c level (OR = 0.152, 95% CI = 0.034–0.674, *P* = 0.013).

## 4. Discussion

The SGLT2i are a newly introduced class of antihyperglycemic agents that were approved for patients with type 2 diabetes in 2013 and 2014 [8]. These drugs lower blood glucose by reducing glucose reabsorption in the renal proximal tubule, and they also induce weight loss and lower blood pressure [21, 22]. Several randomized controlled trials examined their effects on different cardiovascular outcomes [22, 23]. In particular, the EMPA-REG OUTCOME study showed that empagliflozin decreased the rate of hospitalization for heart failure and lowered the rates of cardiovascular and all-cause mortality in patients with established cardiovascular diseases but had no effect on the rates of myocardial infarction or stroke [22]. The CANVAS trial reported that canagliflozin reduced the risk of a composite outcome (cardiovascular death, myocardial infarction, and stroke) by 24% reduced renal complications in those with high risk for cardiovascular diseases but had no effect on myocardial infarction and stroke [23]. The CVD-REAL study, a large multinational study that compared canagliflozin, dapagliflozin, and empagliflozin with other glucose-lowering agents, reported that the use of a SGLT2i was associated with a lower risk of hospitalization for heart failure and all-cause death [24]. Taken together, these previous studies indicate that SGLT2i reduce cardiovascular mortality in patients with type 2 diabetes but have no apparent effect on myocardial infarction and stroke, which is the most common macrovascular complications of diabetes. Furthermore, no previous studies have examined the effect of SGLT2i on DR, which is the major microvascular diabetic complication.

Recent estimates suggest that the number of people with DR will increase dramatically from 127 million in 2010 to 191 million in 2030 [25]. Thus, the burden of DR and blindness must be considered when estimating the socioeconomic burden of type 2 diabetes. Treatment of classic risk factors, such as hyperglycemia and hypertension, can prevent or slow the progression of DR [26]. Laser photocoagulation and intravitreal injections of steroids or anti-VEGF agents can effectively treat complications in patients with preexisting DR, such as diabetic macular edema, vitreous hemorrhage, and proliferative changes [27]. However, these treatments are mainly for patients with late-stage DR and typically cannot provide full restoration of vision [27], so prevention of DR progression is needed to reduce the rate of irreversible complications.

The present investigation of the effect of SGLT2i showed that these agents slowed the progression of DR in patients with type 2 diabetes. The level of HbA1c was higher in patients with SGLT2i compared to control group, but the ratio of patients with DR progression was lower in patients with SGLT2i. We also found that SGLT2i still had a protective effect on DR after matching of patients by glycemic control state (based on HbA1c data) and initial DR grade. The final HbA1c levels also showed no differences between groups. The number of intravitreal anti-VEGF agent injections, which affect DR progression, was not different between groups. This suggests that SGLT2i protected against the progression of DR independently of their effect on lowering of blood glucose.

We did not investigate the mechanism underlying the protective effect of SGLT2i on DR, but other studies suggest possible clues. For example, early-stage DR is characterized by vascular hyperperfusion, with higher blood flow and larger vessel diameters [28–30]. This elevated blood flow can increase shear stress and cause vascular damage, which leads to endothelial dysfunction, disruption of the basement membrane, and remodeling of the extracellular matrix [31]. Recent studies of dapagliflozin reported that an effect independent of glucose lowering was responsible for prevention of arteriole wall thickening, reduction of arterial stiffness [12], reducing oxidative stress, and improving endothelial function [32]. Empagliflozin can also reduce glucotoxicity and oxidative stress and has anti-inflammatory and antifibrotic effects [33, 34].

Metformin is the preferred initial agent for the treatment of type 2 diabetes, and an additional second-line agent is often considered if there is insufficient lowering of HbA1c after 3 months of monotherapy [35]. When prescribing a secondary oral hypoglycemic agent, its effects on vascular complications are an important consideration. We recently reported the association of DR with diastolic dysfunction in type 2 diabetic patients with cardiomyopathy [36], so efforts to prevent the progression of DR might also protect cardiac function. DPP4 inhibitors can protect against DR [16, 37], but their effect on DR remains controversial because they may aggravate vascular leakage [17]. SGLT2i may be a more suitable choice for secondary therapy, because they protect against the progression of DR and also reduce cardiovascular mortality [22, 24].

The major limitations of this study are its retrospective design and the small number of patients. Although we adjusted for confounding factors by propensity score matching, a prospective study with a larger number of patients is needed to confirm the protective effect of SGLT2i on the progression of DR. This study was also limited in that we only examined the progression of preexisting DR; further studies are needed to investigate the effect of SGLT2i on the onset of DR. Nevertheless, this pilot study provides important new information, because it is the first to document the effect of SGLT2i on the progression of DR in a clinical setting.

## 5. Conclusions

Taken together, the present study showed that treatment of type 2 diabetic patients with SGLT2i slowed the progression of DR, and that this protective effect was independent from their glucose-lowering effects. To our knowledge, this is the first study to show that SGLT2i slows the progression of DR. Further prospective randomized double-blind studies are needed to confirm these findings.

## Figures and Tables

**Table 1:** Clinical characteristics of patients in the SGLT2i and control groups before propensity score matching.

	SGLT2i (*n* = 21)	Control (*n* = 71)	*P* value
Age (years)	51.3 ± 9.7	57.8 ± 12.4	0.014^∗^
Sex (male : female)	16 : 5	38 : 33	0.064
Follow-up period (months)	20.1 ± 7.8	25.1 ± 9.2	0.140
Medical history			
Duration of diabetes (years)	11.3 ± 8.9	11.5 ± 9.2	0.963
Presence of hypertension	10/21	37/71	0.717
Presence of CVD	2/21	8/71	0.822
Initial laboratory data			
HbA1c (%)	9.6 ± 2.2	8.2 ± 1.8	0.007^∗^
Total cholesterol (mg/dL)	170.8 ± 45.5	167.4 ± 48.9	0.832
Triglycerides (mg/dL)	181.4 ± 129.7	162.9 ± 159.4	0.148
HDL cholesterol (mg/dL)	48.5 ± 11.6	44.5 ± 11.3	0.168
LDL cholesterol (mg/dL)	91.2 ± 35.3	97.9 ± 52.0	0.669
Last laboratory data			
HbA1c (%)	8.1 ± 1.3	7.6 ± 1.6	0.243
Total cholesterol (mg/dL)	156.3 ± 35.6	156.5 ± 40.9	0.981
Triglycerides (mg/dL)	162.0 ± 90.3	148.8 ± 113.8	0.694
HDL cholesterol (mg/dL)	48.9 ± 9.6	45.9 ± 10.3	0.335
LDL cholesterol (mg/dL)	75.3 ± 25.5	88.7 ± 35.6	0.221
Initial ETDRS score			0.314
20, 35 (mild NPDR)	3	22	
43, 47 (moderate NPDR)	13	29	
53 (severe NPDR)	2	13	
61, 65, 71, 75, 81 (PDR)	3	7	
DR severity (worsened : stable)	3 : 18	31 : 40	0.014^∗^
No. of IVT	0.7 ± 1.2	1.2 ± 1.9	0.368

Data are presented as means ± standard deviations. CVD = cardiovascular disease; DR = diabetic retinopathy; ETDRS = Early Treatment Diabetic Retinopathy Study; HDL = high-density lipoprotein; IVT = intravitreal anti-VEGF injection; LDL = low-density lipoprotein; NPDR = nonproliferative diabetic retinopathy; PDR = proliferative diabetic retinopathy; SGLT2i = sodium-glucose cotransporter 2 inhibitor. ^∗^*P* < 0.05.

**Table 2:** Clinical characteristics of patients in the SGLT2i and control groups after propensity score matching.

	SGLT2i (*n* = 21)	Control (*n* = 21)	*P* value
Age (years)	51.3 ± 9.7	49.4 ± 11.2	0.772
Sex (male : female)	16 : 5	12 : 9	0.190
Follow-up period (months)	20.1 ± 7.8	23.8 ± 13.6	0.512
Medical history			
Duration of diabetes (years)	11.3 ± 8.9	11.0 ± 10.4	0.565
Presence of hypertension	10/21	8/21	0.533
Presence of CVD	2/21	3/21	0.634
Initial laboratory data			
HbA1c (%)	9.6 ± 2.2	9.4 ± 1.9	0.930
Total cholesterol (mg/dL)	170.8 ± 45.5	167.2 ± 45.4	0.798
Triglycerides (mg/dL)	181.4 ± 129.7	136.1 ± 72.6	0.177
HDL cholesterol (mg/dL)	48.5 ± 11.6	44.6 ± 7.2	0.391
LDL cholesterol (mg/dL)	91.2 ± 35.3	100.4 ± 41.4	0.425
Last laboratory data			
HbA1c (%)	8.1 ± 1.3	7.9 ± 1.9	0.804
Total cholesterol (mg/dL)	156.3 ± 35.6	150.1 ± 34.8	0.622
Triglycerides (mg/dL)	162.0 ± 90.3	123.1 ± 46.9	0.153
HDL cholesterol (mg/dL)	48.9 ± 9.6	43.9 ± 7.7	0.131
LDL cholesterol (mg/dL)	75.3 ± 25.5	82.1 ± 26.8	0.516
Initial ETDRS score			0.872
20, 35 (mild NPDR)	3	5	
43, 47 (moderate NPDR)	13	13	
53 (severe NPDR)	2	2	
61, 65, 71, 75, 81 (PDR)	3	1	
DR severity (worsened : stable)	3 : 18	11 : 10	0.009^∗^
No. of IVT	0.7 ± 1.2	1.5 ± 2.2	0.255

Data are presented as means ± standard deviations. CVD = cardiovascular disease; DR = diabetic retinopathy; ETDRS = Early Treatment Diabetic Retinopathy Study; HDL = high-density lipoprotein; IVT = intravitreal anti-VEGF injection; LDL = low-density lipoprotein; NPDR = nonproliferative diabetic retinopathy; PDR = proliferative diabetic retinopathy; SGLT2i = sodium-glucose cotransporter 2 inhibitor. ^∗^*P* < 0.05.

**Table 3:** Logistic regression analysis of the effect of different variables on the progression of DR before propensity score matching in the SGLT2i and control groups.

Variable	OR (95% CI)	*P* value
Age	0.985 (0.951-1.021)	0.411
Sex (female)	1.455 (0.617-3.428)	0.392
Duration of diabetes	0.965 (0.919-1.015)	0.165
Hypertension	0.774 (0.331-1.808)	0.554
CVD	0.705 (0.170-2.929)	0.631
SGLT2i	0.215 (0.058-0.796)	0.021^∗^
HbA1c	1.041 (0.841-1.288)	0.714
Total cholesterol	1.000 (0.991-1.009)	0.944
Triglycerides	0.996 (0.991-1.002)	0.178
HDL cholesterol	0.954 (0.907-1.003)	0.067
LDL cholesterol	1.004 (0.991-1.017)	0.589

Data are presented as odd ratios (95% confidence interval). CVD = cardiovascular disease; HDL = high-density lipoprotein; LDL = low-density lipoprotein; SGLT2i = sodium-glucose cotransporter 2 inhibitor. ^∗^*P* < 0.05.

**Table 4:** Logistic regression analysis of the effect of different variables on the progression of DR after propensity score matching in the SGLT2i and control groups.

Variable	OR (95% CI)	*P* value
Age	0.978 (0.917-1.043)	0.500
Sex (female)	1.173 (0.304-4.527)	0.817
Duration of diabetes	1.007 (0.944-1.073)	0.835
Hypertension	0.236 (0.054-1.035)	0.056
CVD	1.389 (0.204-9.445)	0.737
SGLT2i	0.152 (0.034-0.674)	0.013^∗^
HbA1c	1.287 (0.916-1.809)	0.146
Total cholesterol	0.997 (0.982-1.012)	0.665
Triglycerides	0.980 (0.960-1.000)	0.050
HDL cholesterol	0.936 (0.854-1.026)	0.159
LDL cholesterol	1.003 (0.981-1.025)	0.808

Data are presented as odd ratios (95% confidence interval). CVD = cardiovascular disease; HDL = high-density lipoprotein; LDL = low-density lipoprotein; SGLT2i = sodium-glucose cotransporter 2 inhibitor. ^∗^*P* < 0.05.

## Data Availability

The data used to support the findings of this study are included within the article.
